# Analysis of the spike, ORF3, and nucleocapsid genes of porcine epidemic diarrhea virus circulating on Thai swine farms, 2011–2016

**DOI:** 10.7717/peerj.6843

**Published:** 2019-04-30

**Authors:** Supansa Tuanthap, Sompong Vongpunsawad, Cherdpong Phupolphan, Ausanee Duang-in, Suphot Wattanaphansak, Pornchalit Assavacheep, Apiradee Theamboonlers, Supol Luengyosluechakul, Alongkorn Amonsin, Yong Poovorawan

**Affiliations:** 1 Interdisciplinary Program of Biomedical Sciences, Graduate School, Chulalongkorn University, Bangkok, Thailand; 2 Center of Excellence in Clinical Virology, Faculty of Medicine, Chulalongkorn University, Bangkok, Thailand; 3 The Livestock Animal Hospital, Faculty of Veterinary Science, Chulalongkorn University, Nakhon Pathom, Thailand; 4 Department of Veterinary Medicine, Faculty of Veterinary Science, Chulalongkorn University, Bangkok, Thailand; 5 Center of Excellence for Emerging and Reemerging Infectious Diseases in Animals, Faculty of Veterinary Science, Chulalongkorn University, Bangkok, Thailand

**Keywords:** Pig, Porcine epidemic diarrhea virus, Spike, ORF3, Nucleocapsid, Thailand

## Abstract

Porcine epidemic diarrhea virus (PEDV) outbreaks on pig farms have caused significant economic loss in the swine industry since it was first reported in Thailand a decade ago. Anecdotal evidence suggests that PEDV is now endemic in this region, therefore genome information of circulating PEDV is important for molecular surveillance and evaluation of potential benefits of field vaccination. Here, we characterized PEDV infection on commercial Thai swine farms by screening 769 samples of feces and small intestinal contents from pigs with diarrhea between 2011 and 2016. Using reverse-transcription polymerase chain reaction targeting the spike (S) gene, 153 PEDV-positive samples were further subjected to analysis of the open reading frame 3 and nucleocapsid (N) genes. Comparison of 95 samples in which nucleotide sequencing was successfully obtained for all three genes revealed evolutionary diversity among the Thai PEDV strains. Phylogenetic analyses suggest that although some Thai strains changed little from years past, others resembled more closely to the recent strains reported in China. Interestingly, eight Thai PEDV strains possessed amino acid deletions in the N protein. The PEDV sequence divergence may be responsible for driving periodic outbreaks and continued persistence of PEDV on commercial swine farms. Our findings provide important insight into regional PEDV strains in circulation, which may assist future inclusions of suitable strains for future PEDV vaccines.

## Introduction

Porcine epidemic diarrhea virus (PEDV) contributes to enteropathogenic diarrhea, especially among suckling piglets, and causes significant economic loss to the swine industry worldwide (Choudhury et al., 2016; Jung & Saif, 2015). PEDV-infected pigs experience watery diarrhea, vomiting and severe dehydration, which leads to 50–90% mortality among susceptible piglets (Madson et al., 2014; Shibata et al., 2000). Transmission is frequently fecal-oral, but infections via airborne and fomites have been documented (Alonso et al., 2014; Lowe et al., 2014; Sasaki et al., 2016).

Porcine epidemic diarrhea virus is a single-stranded positive-sense RNA virus belonging to the family *Coronaviridae* and the genus *Alphacoronavirus* (Lin et al., 2016). PEDV genome encodes the replicase polyprotein 1a and 1b (processed into 16 non-structural proteins nsp1–nsp16), spike (S), open reading frame 3 (ORF3), envelope (E), membrane (M), and nucleocapsid (N) (Song & Park, 2012). The most widely-accepted classification of PEDV is based on the S gene sequence, which categorizes PEDV genotypes into two genogroups (G1 and G2). Each genogroup is further sub-divided into subgroups (G1a, G1b, G2a, and G2b). Classical strains are designated G1a, while the new variant strains (S INDEL) strains belong to G1b. The highly virulent Asian and North American strains are designated G2a and G2b, respectively (Lin et al., 2016). The original PEDV (represented by the prototypic CV777 strain) was first reported in the 1970s in Europe and was associated with high morbidity and mortality among infected pigs (Pensaert & De Bouck, 1978).

The S INDEL strains were first reported in the U.S. in 2014 and were subsequently introduced to Asia and Europe. These strains contain insertions and deletions in the N-terminal region of the S protein (S1 region) compared to the prototype CV777. One-third of the S gene among the new variants G1b shared greater than 95% identity to the classical G1a strain, while the remaining two-thirds possessed greater than 99% similarity to highly virulent G2 strains. Therefore, S INDEL variants resemble recombination strains between G1a and G2 (Huang et al., 2013; Wang et al., 2014). Infection by some S-INDEL variants was reported to produce decreased symptom severity including moderate diarrhea, lower titers of viral shedding, and reduced mortality (Murakami et al., 2015; Vlasova et al., 2014). The highly virulent G2 strains caused an epidemic in Asia and have been identified in North America and elsewhere around the world (Lee, 2015).

Thailand has a major pork production industry in Southeast Asia. Despite improved animal husbandry, farm management, vaccination, and boosting of lactogenic immunity, PEDV outbreaks continue to occur on Thai swine farms. Loss of piglets due to PEDV infection necessitates constant epidemiological surveillance to effectively monitor transmission. To determine the genetic relationship among the current and past PEDV strains in Thailand compared to the global strains, we characterized the S, ORF3, and N genes and evaluated the deduced amino acid sequence variations in 95 PEDV strains from commercial swine farms throughout the country.

## Materials and Methods

### Samples

A total of 769 samples were submitted to the Livestock Animal Hospital of the Faculty of Veterinary Science, Chulalongkorn University in Nakhon Pathom province between May 2011 and August 2016. These represent archived and convenient samples from 123 commercial pig farms located throughout Thailand (Fig. S1). Most of the samples were primarily submitted from the central part of Thailand where the majority of swine farms are located. Samples were from western provinces (Kanchanaburi, Prachuap Khiri Khan, Phetchaburi, and Ratchaburi; *n* = 316), central provinces (Lop Buri, Samut Songkhram, Suphan Buri, Saraburi, Phra Nakhon Si Ayutthaya, and Nakhon Pathom; *n* = 173), eastern provinces (Chon Buri and Chachoengsao, *n* = 109), northeastern provinces (Ubon Ratchathani, Udon Thani, and Nakhon Ratchasima, *n* = 80), southern provinces (Trang and Nakhon Si Thammarat, *n* = 26), and from unspecified locations (*n* = 65).

Feces (*n* = 509) and small intestinal content (intestinal mucosa of the duodenum and upper part of jejunum from tissues scraping, *n* = 260) from diarrheic pigs were prepared as 10% (w/v) suspension in sterile phosphate buffered saline. Clarified filtrates were collected after centrifugation at 3,000×*g* for 20 min. The Institutional Animal Care and Use Committee (IACUC number 1731020) and the Institutional Biosafety Committee (IBC number 1731008) of Chulalongkorn University approved this study.

### Reverse-transcription polymerase chain reaction

Viral RNA was extracted using Ribospin vRD II viral RNA purification Kit (GeneAll, Seoul, Korea) according to manufacturer’s instructions. Partial S gene was amplified by RT-PCR using SuperScript III One-Step RT-PCR with Platinum Taq DNA polymerase (Invitrogen, Carlsbad, CA, USA) as previously described (Kim, Song & Park, 2001). RT was performed at 48 °C for 45 min. PCR cycling parameters were initial denaturation at 95 °C for 2 min, followed by 30 cycles of denaturation at 94 °C for 30 s, annealing at 55 °C for 1 min, extension at 72 °C for 1 min, and a final extension at 72 °C for 5 min. Samples tested positive for the S gene were subjected to the amplification of ORF3 and the N gene using annealing temperatures of 51 °C and 55 °C, respectively (Table S1). Amplicons were purified using agarose gel electrophoresis and subjected to Sanger sequencing. Nucleotide sequences of the Thai strains obtained from this study were deposited in the GenBank database (accession numbers are in Table S2; raw sequence data are in Datasets S1–S3).

### Nucleotide and amino acid sequence analyses

Nucleotide sequences were assembled and edited using SeqMan II and aligned using BioEdit and ClustralX. Genetic relatedness among the Thai PEDV strains from this study (*n* = 95) was compared to previously identified Thai strains, global strains, and PEDV vaccine strains. Phylogenetic trees were constructed using the maximum likelihood method and 1,000 bootstrap replicates implemented in MEGA6 (Tamura et al., 2013). Best model fitting was automatically calculated for genetic distances (HKY+G model for S gene, T93+G model for ORF3, and K2+G model for N gene). Bootstrap values ≥80% were considered significant. Vaccine strains in which sequences were available for inclusion in all three phylogenetic trees were CV777 (Belgian strain), attenuated DR13 (Korean strain), and 96P4-C6 (Japanese strain). Deduced amino acid sequences of the Thai strains were also compared to those of the vaccine strains and reported as amino acid identity unless otherwise stated. Residue position numbering was based on CV777.

## Results

The partial S gene primers flanking the CO-26K equivalent (COE) domain were used to initially screen all samples for PEDV. COE domain is one of several epitope regions with high variations, which may affect viral neutralization and reflect PEDV genetic diversity. In all, 153 out of 769 samples tested positive for the PEDV S gene. Among these, 95 samples yielded sufficient amplification products for further sequence analysis of the partial S, ORF3, and N genes. PEDV-positive samples were derived from animals aged between 3 days and 8 weeks. A 15-week old pig and two lactating sows were the only three exceptions. PEDV was most prevalent in the central part of Thailand where many pig farms are located (Fig. S2; Table S3).

### Analysis of the S gene

**Figure 1:**
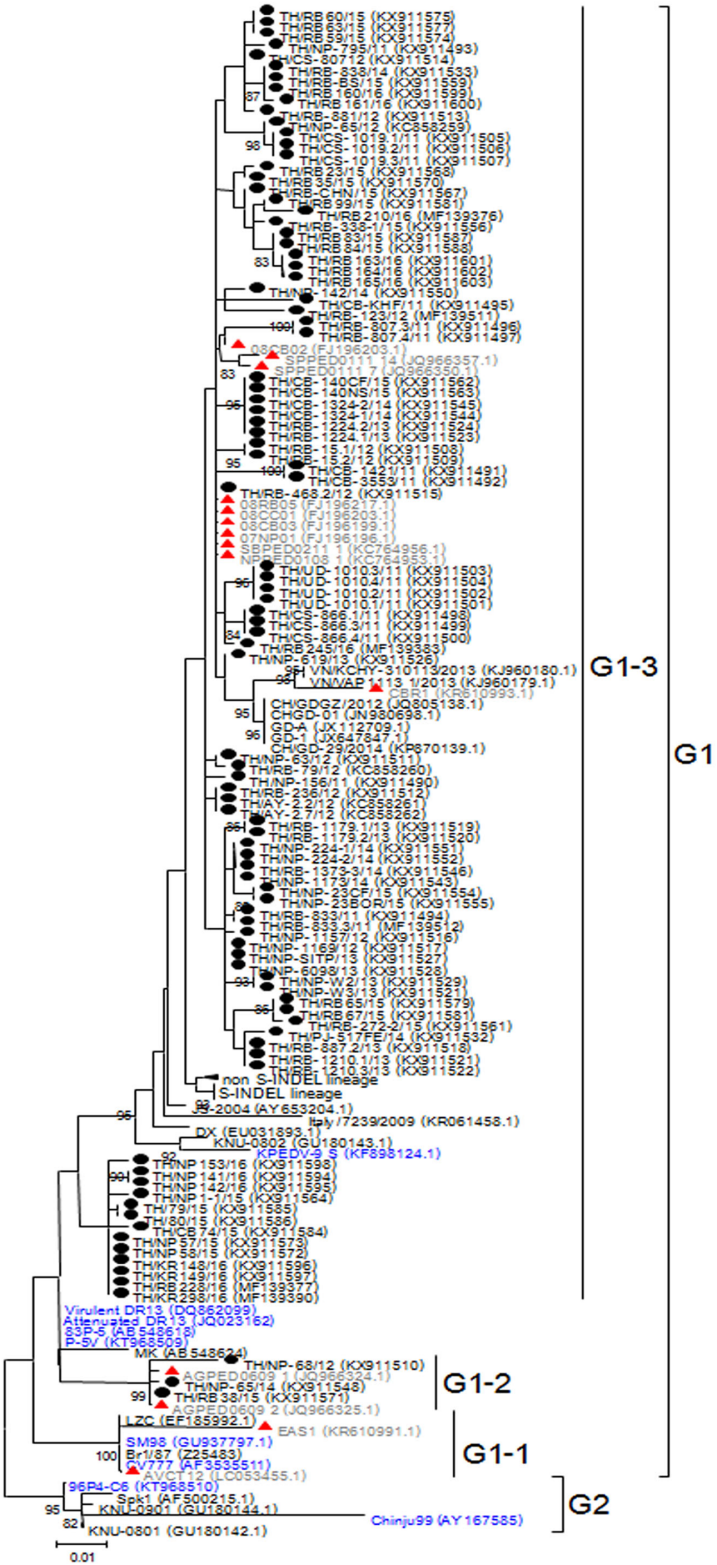
Phylogenetic analysis of the partial S gene. Previously identified PEDV in Thailand from past years (red) and 95 strains identified in this study (black) were compared to the vaccine strains (blue) using the maximum likelihood method implemented in MEGA6. Bootstrap values ≥80% from 1,000 replicates are shown at the nodes. Strain names are followed by the accession numbers in parentheses.

Based on genetic analysis, the S sequences clustered into two major groups (Fig. 1). While the historical CV777 strain was assigned in the G1-1 group, the majority (92/95) of the Thai PEDV strains in this study either clustered with previously identified Thai strains or with some of the common vaccine strains such as KPEDV-9 and attenuated DR13 in the G1-3 group. Three Thai PEDV strains (NP-68/12, NP-65/14, and RB38/15) were genetically distinct from others and formed a G1-2 cluster with Thai PEDV strains AGPED0609_1, AGPED0609_2 previously identified in 2011.

The deduced amino acid residues of the S protein showed that Thai PEDV strains consistently shared amino acid sequence identity to the PEDV strains reported on swine farms in Vietnam (92.3–99.4% identity), Japan (93.8–98.7% identity), China (92.8–99.2% identity), Korea (91.9–99.4% identity), and the U.S. (93.6–98.7% identity). Moreover, the Thai strains shared 92.8–96.6% identity to the prototypic strain CV777 and 93.7–98.5% identity to other vaccine strains (Table S4). We note that one Thai PEDV strain designated RB-468.2/12 shared 100% identity to Thai strains from other studies (08RB05, 08CC01, 08CB03, 07NP01, SBPED0211_1, and NPPED0108_1), all of which were previously identified in or before 2011.

Alignment of amino acid sequences encompassing positions 507–689 revealed that all 95 Thai strains differed from CV777 at residues S523G, V527I, and I635V. Additionally, all 92 Thai strains from the G1-3 group shared G594S, most of which also possessed A517S, L521H, T549S, L612F, and I667F. The three G1-2 Thai strains were primarily characterized by S567G and M641I, of which NP-68/12 differed most from CV777 at 14 residues over this region (Figs. S3A–S3D).

### Analysis of the ORF3 gene

**Figure 2:**
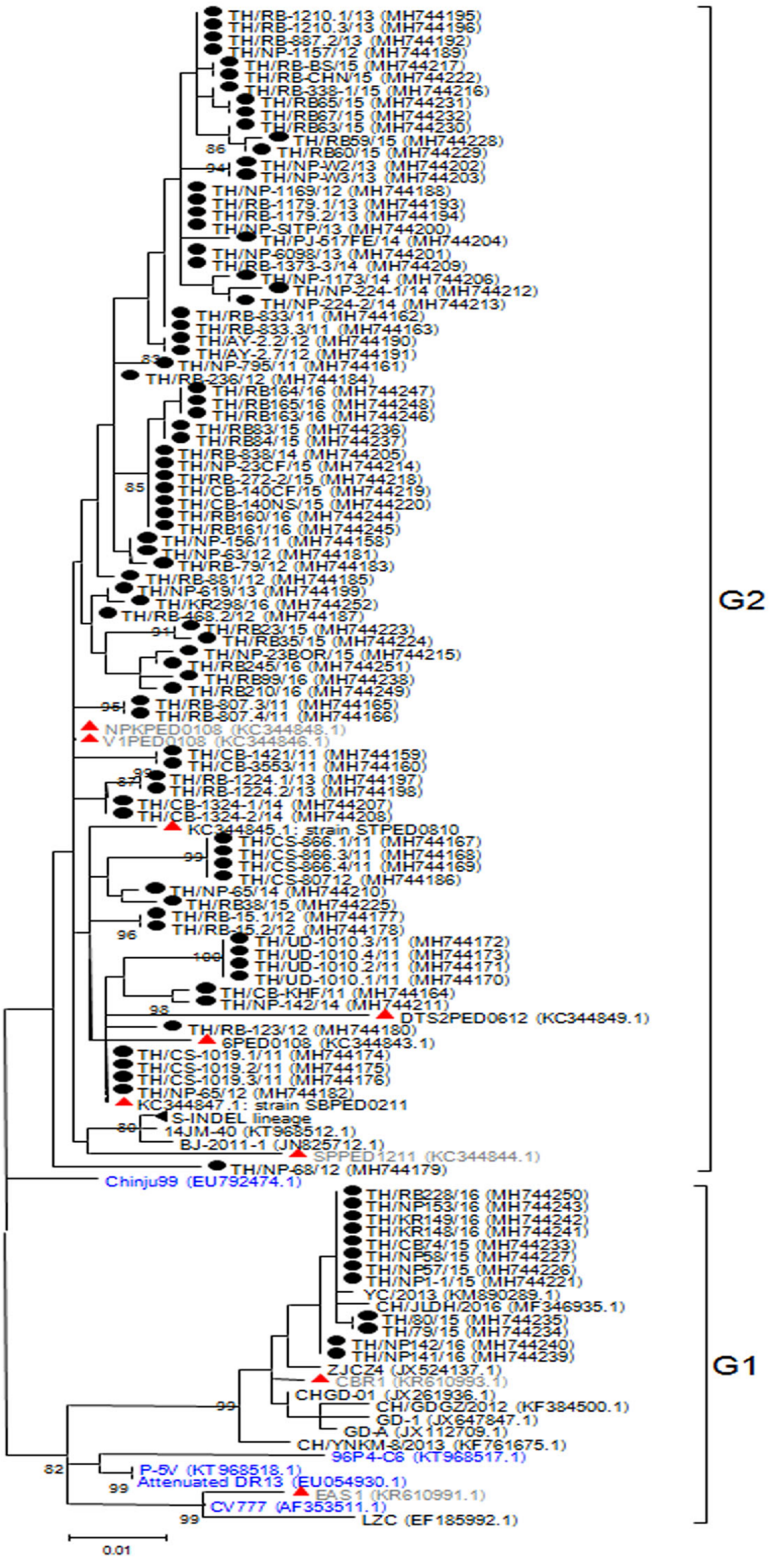
Phylogenetic analysis of the complete ORF3 gene. Previously identified PEDV in Thailand from past years (red) and 95 strains identified in this study (black) were compared to the vaccine strains (blue) using the maximum likelihood method implemented in MEGA6. Bootstrap values ≥80% from 1,000 replicates are shown at the nodes. Strain names are followed by the accession numbers in parentheses.

The ORF3 gene was phylogenetically divided into two groups. A number of the Thai PEDV strains (12/95) belonged to the G1 group with CV777 and the attenuated DR13 strain (Fig. 2). The majority of the Thai strains (83/95) were more closely related to the previous Thai strains than to the vaccine strains and were grouped in G2. The S-INDEL strains were also in this group. Deduced amino acid sequences showed that the Thai PEDV strains shared 94.4–100% amino acid identity with each other, 95.8–97% identity to CV777, and 95–98.1% identity to the other vaccine strains. In particular, Thai G1 strains in this study shared 99.7–99.8% identity to a Chinese YC/2013 strain from 2013, and 98.8–99.1% identity to a previous Thai strain CBR1 identified in 2014. Meanwhile, the Thai G2 strains in this study shared 95.8–100% identity to previous Thai strains from 2008 to 2012. All Thai strains were similar to CV777 in their absence of deletions of residues 82–99 and 138–139 in the ORF3 protein compared to other vaccine strains (Table S5).

### Analysis of the N gene

**Figure 3:**
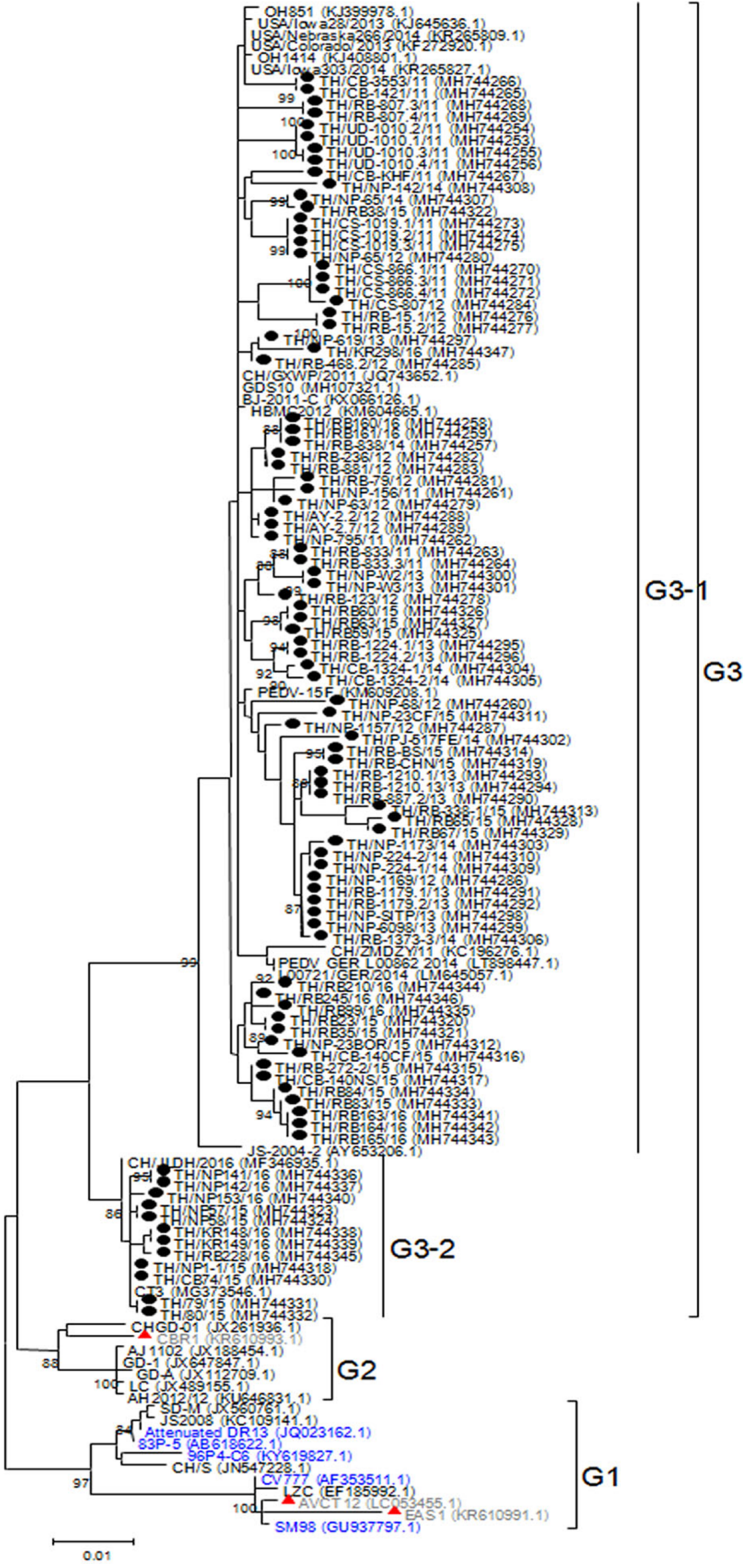
Phylogenetic analysis of the partial N gene. Previously identified PEDV in Thailand from past years (red) and 95 strains identified in this study (dotted) were compared to the vaccine strains (blue) using the maximum likelihood method implemented in MEGA6. Bootstrap values ≥80% from 1,000 replicates are shown at the nodes. Strain names are followed by the accession numbers in parentheses.

While CV777 and other vaccine strains clustered within the G1 group, all Thai PEDV strains in this study appeared to have diverged and branched off as a separate group (Fig. 3). Although they shared 96–100% amino acid identity, the Thai strains belonging to G3-1 (83/95) are genetically close to the reference strains previously identified in the U.S. (OH851) and China (CH/ZMDZY/11) (97.1–99.7% amino acid identity). In the G3-2 group, the Thai strains TH/NP1-1/15 and TH/CB74/15 showed identical nucleotide sequences to the Vietnamese strain CT3. We next analyzed the deduced amino acid sequences of the N protein from the Thai strains, which spanned residues 13–406 (out of 441 residues). Sequence alignment with CV777 showed that eight Thai strains possessed several residue deletions in the middle of the N protein encompassing positions 241–251 in addition to several residue differences (Fig. S4). Four Thai strains (TH/UD-1010.1/11, TH/UD-1010.2/11, TH/UD-1010.3/11, TH/UD-1010.4/11) were missing residues R241 and H242. Three Thai strains (TH/RB160/16, TH/RB161/16, and TH/RB-838/14) were missing residues K243 and Q244. Finally, residue E251 was absent in TH/NP-68/12.

## Discussion

Improvement in the molecular epidemiology of PEDV has advanced the understanding of viral transmission and spread. Most studies now involve the characterization of multiple gene sequences, namely S, M, ORF3, and even full-length genome analysis (Cheun-Arom et al., 2016; Puranaveja et al., 2009; Stott et al., 2017; Temeeyasen et al., 2014). The analysis of the S sequence from strains in the U.S. between 2016 and 2017 has identified three new emergent strains (termed S1 NTD-del PEDV variants). These variants possessed several large deletions within S1 N-terminal domain and differed from the original U.S. S INDEL (Su et al., 2018). High variation region on the antigenic COE domain seen in the Thai PEDV strains identified between 2011 and 2016 showed that PEDV in Thailand continue to evolve. The COE domain was first identified in the transmissible gastroenteritis virus and is a known PEDV neutralizing epitope (Chang et al., 2002; Hou et al., 2017). As this region is under immune pressure from the host, sequence variations are often found throughout this most distal region of the S protein. It was not surprising that distinct groups of Thai strains (namely G1-2 and G1-3) were circulating on Thai farms, both of which varied from the vaccine strains. As a result, variations on the epitope region seen in circulating PEDV strains may affect viral neutralization and vaccine escapes. Our observation that the vaccine strains are phylogenetically distant from the majority of the circulating PEDV strains might reflects the relatively low vaccine efficacy afforded by some of the current vaccine strains, of which CV777, 83P-5, SM98, and DR13 have been used in attenuated vaccines in Asia (Gerdts & Zakhartchouk, 2017; Song, Moon & Kang, 2015). However, prediction of viral antigenicity should not rely solely on the variations on the COE antigenic epitopes, but should also depend on the other epitope region variations, serological assays, and/or animal experiment studies.

Open reading frame 3 is a 224 amino acid accessory and a transmembrane protein, which possesses a potassium ion channel function (Teeravechyan et al., 2016). It is known that the ORF3 gene product is not required for viability as PEDV generated from cDNA clones lacking the ORF3 is infectious (Jengarn et al., 2015). Our analysis of the entire ORF3 gene showed that Thai strains clustered with the vaccine strains. In contrast to the field strains, several vaccine strains including the attenuated DR13, KPEDV-9, and P-5V have a 51 nucleotide deletion between position 245 and 295 in their ORF3 gene (Li et al., 2014). This notable sequence difference may have contributed to the clustering of the phylogenetic tree of ORF3 into two groups.

Previous reports of molecular surveillance of PEDV on Thai swine farms have mainly examined the genetic diversity of the S, M, and ORF3 genes. Less frequently examined is the N gene because its crucial function in binding viral RNA genome partially constrains changes in its sequence (Puranaveja et al., 2009; Temeeyasen et al., 2014). The N protein is abundantly expressed during the early stages of PEDV infection and is therefore a preferred target for PEDV detection. Sequence variations on the N gene could also be used to evaluate PEDV genetic diversity and relationship among circulating PEDV strains. Our phylogenetic analysis of the near-complete N gene showed that the Thai strains genetically grouped with the recent PEDV strains from the U.S. and China. Meanwhile, the vaccine strains were more genetically distant compared to the current field strains in circulation. Interestingly, eight Thai PEDV strains demonstrated up to two residue deletions in the middle of the N protein. These missing residues encompassing position 241–251 have not been previously characterized in other PEDV or alphacoronaviruses even though the N gene has the greatest sequence similarities to TGEV and human coronaviruses (HCV 229E and HCV NL63) (Bridgen et al., 1993; Lin et al., 2015). This observation was reproducible by repeating the RNA extraction of samples, amplification, and sequencing. In addition, samples came from different farms and provinces, and therefore are unlikely to be due to artifacts or contamination. These deletions involved residues located within the immunodominant region of the N protein between residues 136 and 289 previously shown to be associated with NF-ĸB activation pathway (Cao et al., 2015). Residue deletions in this region observed in some Thai PEDV strains, therefore, may indicate an evolving flexible domain with no major functional significance. Nevertheless, pathological relevance of this deletion requires further examination and will benefit from reverse genetic studies.

In this study, several Thai PEDV strains from animals of different age, herd, and year of collection had identical amino acid sequences and belonged to the same subtypes (Table S6). This observation is consistent with the potential transmission of PEDV from farm to farm due to animal transportation and trade (Beam et al., 2015; Puranaveja et al., 2009). The recurrent PEDV outbreaks on Thai farms were also likely due to circulation and re-emergence within the swine herds as a result of the common practice of restocking new susceptible pigs with improper gilt acclimatization, insufficient colostrum intake in newborn piglets, and insufficient immunization of pregnant sow. Alternatively, older pigs with asymptomatic PEDV infection may serve as a reservoir and spread the infection sub-clinically. Vertical transmission is also possible as PEDV RNA has been detected in the milk of infected lactating sows (Li et al., 2012; Sun et al., 2012).

Based on the phylogenetic analysis of the partial S gene, most of the Thai strains in this study belonged to the high virulent G2a lineage. Several Thai strains (23/95), especially those from 2013 to 2015, were genetically related to the new variant G2b strains (Chinese-like strains and U.S.-like strains). We found that the circulating PEDV strains in Thailand continue to diverge from the classical vaccine strains such as the attenuated DR13, CV777, and 96P4-C6. This underscores a major obstacle in the control of disease transmission. Although some vaccines are derived from high-passaged laboratory cultivated viral stocks and are administered orally or intramuscularly to pregnant sows, there is rarely a complete protection among nursing piglets from subsequent PEDV infection (Jung & Saif, 2015). Furthermore, induction of immunity among lactating sows from ingestion of fecal slurry or homogenized intestines of infected neonatal piglets presents the risk of unintended transmission of other enteric pathogens.

This study has several limitations. We did not include in our analysis the PEDV strains with missing S, ORF3 or N gene sequences, which could have revealed additional diversity of PEDV strains circulating in Thailand. Samples in our study were primarily submitted from pigs in central Thailand where the majority of swine farms are located, therefore surveillance of farms in other parts of the country was incomplete. Nevertheless, our multi-year study provides additional knowledge regarding the diversity of PEDV in this region and may assist in determining suitable PEDV strains for future vaccine development.

## Conclusion

Assessing the diversity of PEDV on Thai swine farms was facilitated by the analysis of the S gene in combination with ORF3 and the N genes. Circulating PEDV strains differed significantly from the vaccine strains, which may explain vaccine failure in the field. Continued molecular epidemiology and surveillance will be important in monitoring PEDV transmission.

## Supplemental Information

10.7717/peerj.6843/supp-1 Supplemental Information 1 Farm locations and provincial origins of 769 samples in this study.

10.7717/peerj.6843/supp-2 Supplemental Information 2 The percentage detection and provincial origins of 95 PEDV strains. The percentage detection of each provinces are determined and represent in pie chart. NP, Nakhon Pathom; RB, Ratchaburi; CS, Chachoengsao; CB, Chon Buri; UD, Udon Thani; AY, Phra Nakhon Si Ayutthaya; KR, Kanchanaburi; and PJ, Prachuap Khiri Khan.

10.7717/peerj.6843/supp-3 Supplemental Information 3 Amino acid sequence alignment of the partial S gene encompassing the COE domain of the Thai PEDV strains and the prototypic CV777. Numbers indicate residue position. Identical residues are dotted. Strain NP-68/12 differed most from CV777 which showed in gray highlight.

10.7717/peerj.6843/supp-4 Supplemental Information 4 Amino acid sequence alignment of the partial S gene encompassing the COE domain of the Thai PEDV strains and the prototypic CV777. Numbers indicate residue position. Identical residues are dotted. Strain NP-68/12 differed most from CV777 which showed in gray highlight.

10.7717/peerj.6843/supp-5 Supplemental Information 5 Amino acid sequence alignment of the partial S gene encompassing the COE domain of the Thai PEDV strains and the prototypic CV777. Numbers indicate residue position. Identical residues are dotted. Strain NP-68/12 differed most from CV777 which showed in gray highlight.

10.7717/peerj.6843/supp-6 Supplemental Information 6 Amino acid sequence alignment of the partial S gene encompassing the COE domain of the Thai PEDV strains and the prototypic CV777. Numbers indicate residue position. Identical residues are dotted. Strain NP-68/12 differed most from CV777 which showed in gray highlight.

10.7717/peerj.6843/supp-7 Supplemental Information 7 Amino acid sequence alignment of the partial N gene from Thai PEDV strains with deletions. Numbers indicate residue position. Identical residues are dotted. Deletions are noted with dashed lines covered by the red boxes.

10.7717/peerj.6843/supp-8 Supplemental Information 8 Nucleotide sequence primers. Nucleotide primers used to amplify the PEDV genes.

10.7717/peerj.6843/supp-9 Supplemental Information 9 Nucleotide accession numbers of the Thai PEDV strains.

10.7717/peerj.6843/supp-10 Supplemental Information 10 The details of provincial origin and year collection of 95 PEDV strains. NP, Nakorn Pathom; RB, Ratchaburi; CS, Chachoengsao; CB, Chonburi; UD, Udon Thani; AY, Phra Nakhon Si Ayutthaya; PJ, Prachuap Khiri Khan; NR, Nakhon Ratchasima; and n/a, unknown farm location.

10.7717/peerj.6843/supp-11 Supplemental Information 11 Comparison of amino acid identities between the Thai PEDV and the vaccine strains. The COE domain of Thai PEDVs had the most sequence variation compared to vaccine strains, while ORF3 and N genes are more conserved.

10.7717/peerj.6843/supp-12 Supplemental Information 12 Residue differences within the S, ORF3, and N of the Thai PEDV, CV777, and other vaccine strains. Vaccine strains include Attenuated DR13, 94P4-C6, and P-5V (strain KPEDV-9, 83P-5 were excluded because their sequence not available).

10.7717/peerj.6843/supp-13 Supplemental Information 13 The subtypes of 95 Thai PEDV strains. S gene is sub-divided into subgroup 1 and 3, subgroup 1 and 2 for N gene, whereas major groups of ORF3 gene are not sub-divided into subgroup.

10.7717/peerj.6843/supp-14 Supplemental Information 14 Spike gene sequences. Spike gene sequence with accession number of 95 PEDV strains.

10.7717/peerj.6843/supp-15 Supplemental Information 15 ORF3 gene sequences. ORF3 gene sequence with accession number of 95 PEDV strains.

10.7717/peerj.6843/supp-16 Supplemental Information 16 Nucleocapsid gene sequences. Nucleocapsid gene sequence with accession number of 95 PEDV strains.

## Additional Information and Declarations

### DNA Deposition

The following information was supplied regarding the deposition of DNA sequences: The DNA sequences are available in the Genbank database (https://www.ncbi.nlm.nih.gov/). The accession numbers are listed below: for S gene – MF139511–MF139512 – MF139376–MF139377 – MF138383 – KC858259–KC858262 – KX911490–KX911510 – KX911512–KX911530 – KX911532–KX911533 – KX911543–KX911546 – KX911548 – KX911550–KX911552 – KX911554–KX911556 – KX911559 – KX911561–KX911564 – KX911567–KX911568 – KX911570–KX911571 – KX911573–KX911575 and MH744158–MH744346 for ORF3 and N genes.

### Data Availability

The following information was supplied regarding data availability: The raw data of 3 gene sequences and their accession numbers are available in Datasets S1–S3.
