# Supplementary figures and images for: Analysis of the spike, ORF3, and nucleocapsid genes of porcine epidemic diarrhea virus circulating on Thai swine farms, 2011–2016
Source: PeerJ. 2019 Apr 30;7:e6843. doi: 10.7717/peerj.6843 (PMC6499054; doi:10.7717/peerj.6843)

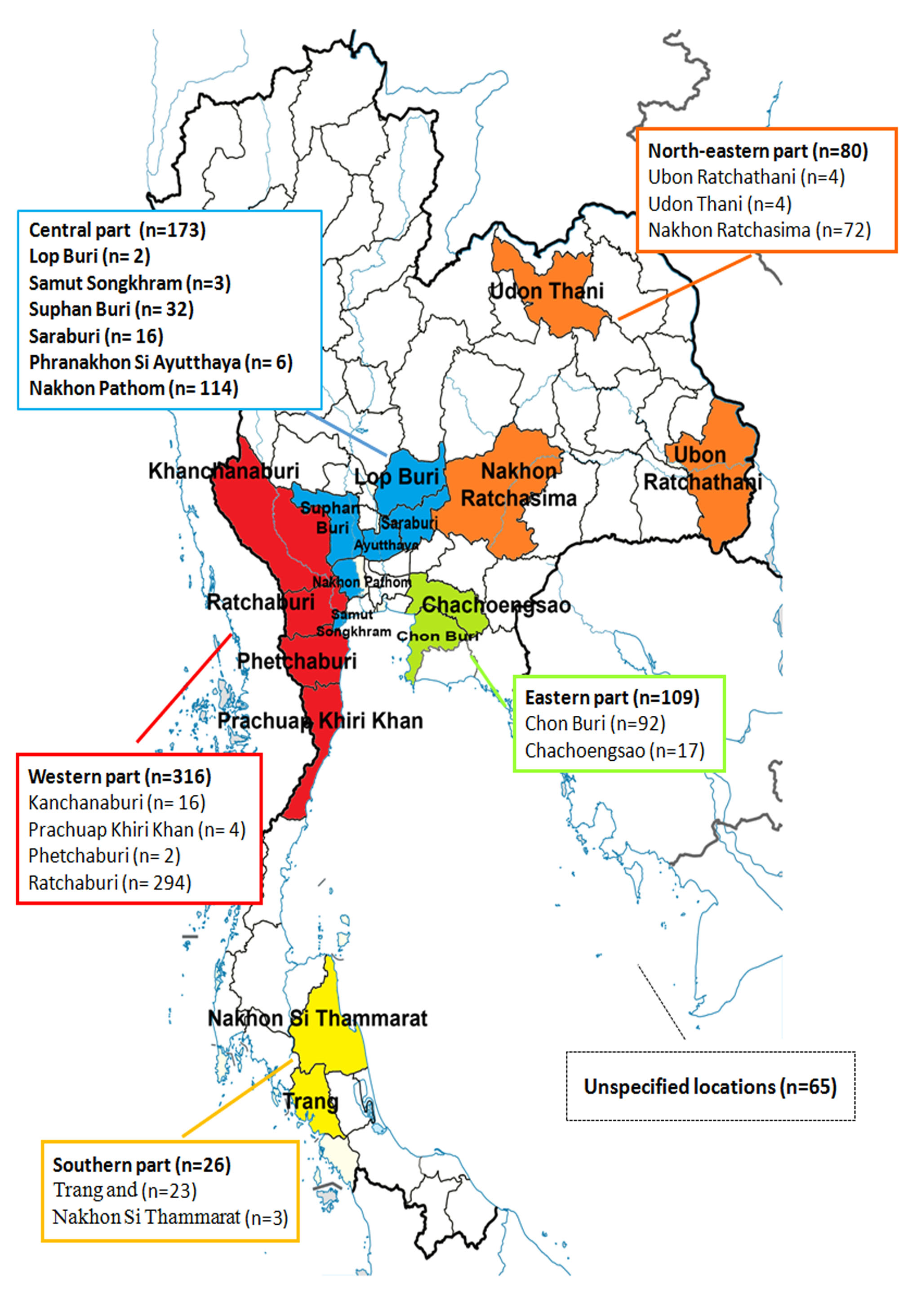

Supplement: Supplemental Information 1 [file peerj-07-6843-s001.png]

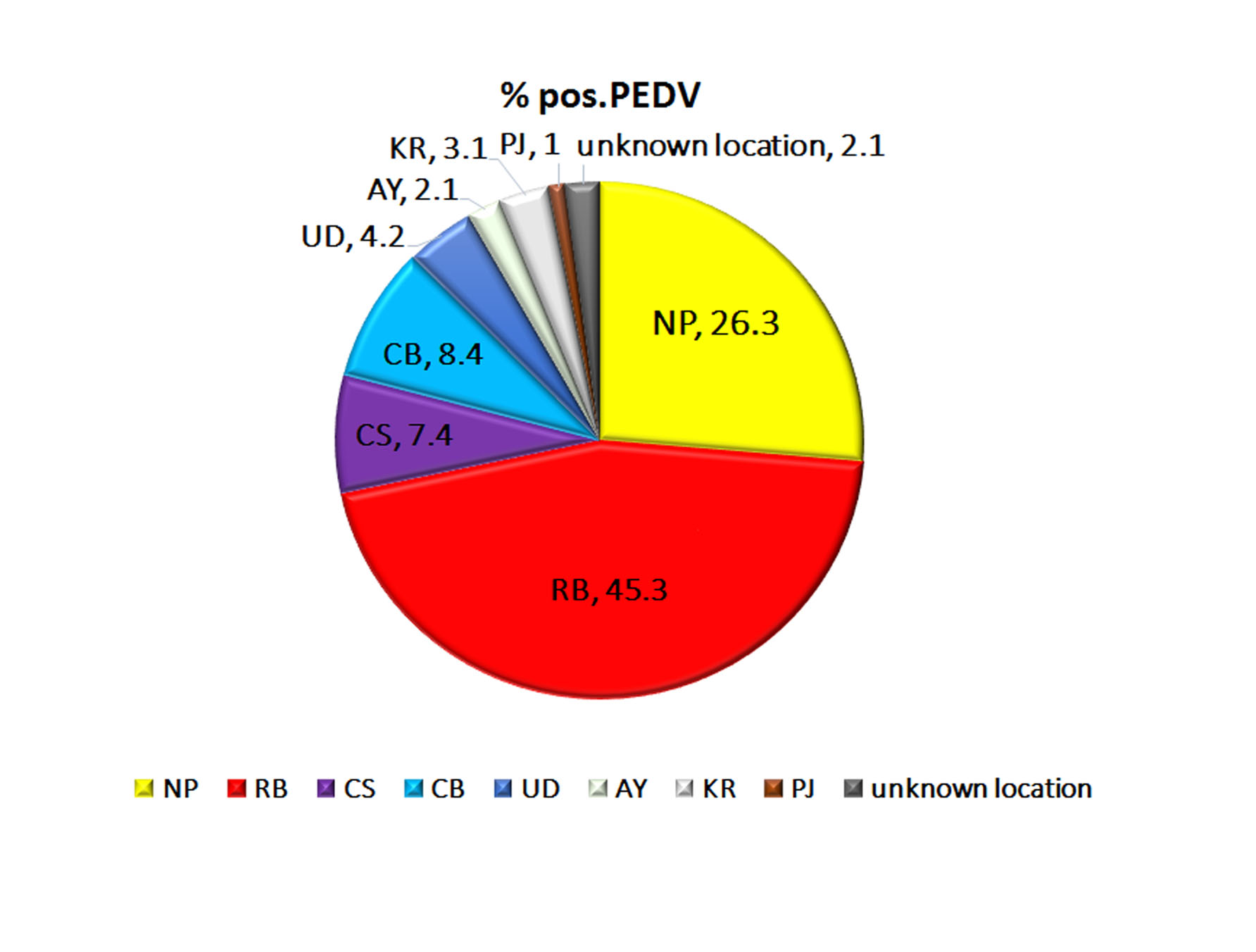

Supplement: Supplemental Information 2 — The percentage detection of each provinces are determined and represent in pie chart. NP, Nakhon Pathom; RB, Ratchaburi; CS, Chachoengsao; CB, Chon Buri; UD, Udon Thani; AY, Phra Nakhon Si Ayutthaya; KR, Kanchanaburi; and PJ, Prachuap Khiri Khan. [file peerj-07-6843-s002.png]

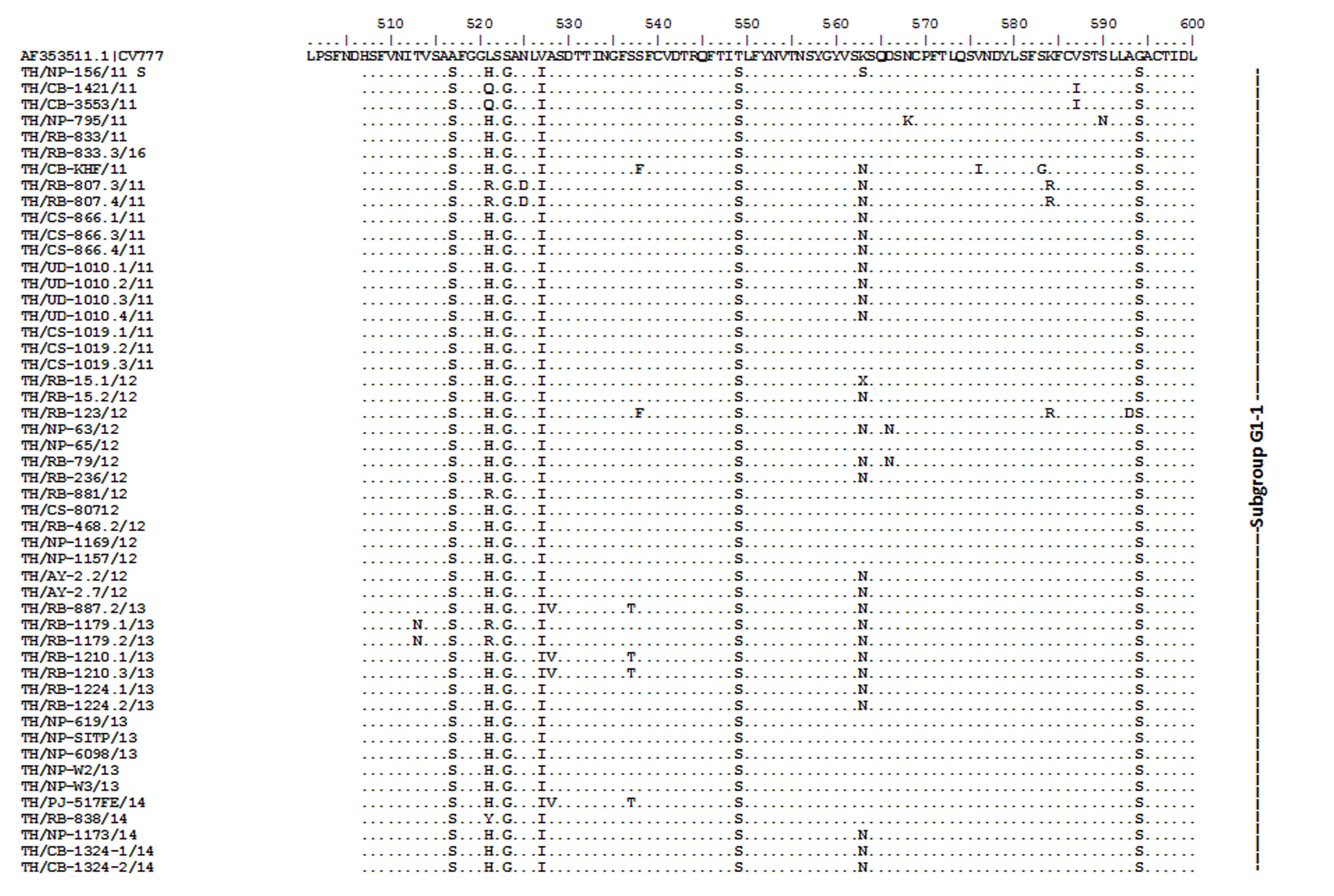

Supplement: Supplemental Information 3 — Numbers indicate residue position. Identical residues are dotted. Strain NP-68/12 differed most from CV777 which showed in gray highlight. [file peerj-07-6843-s003.png]

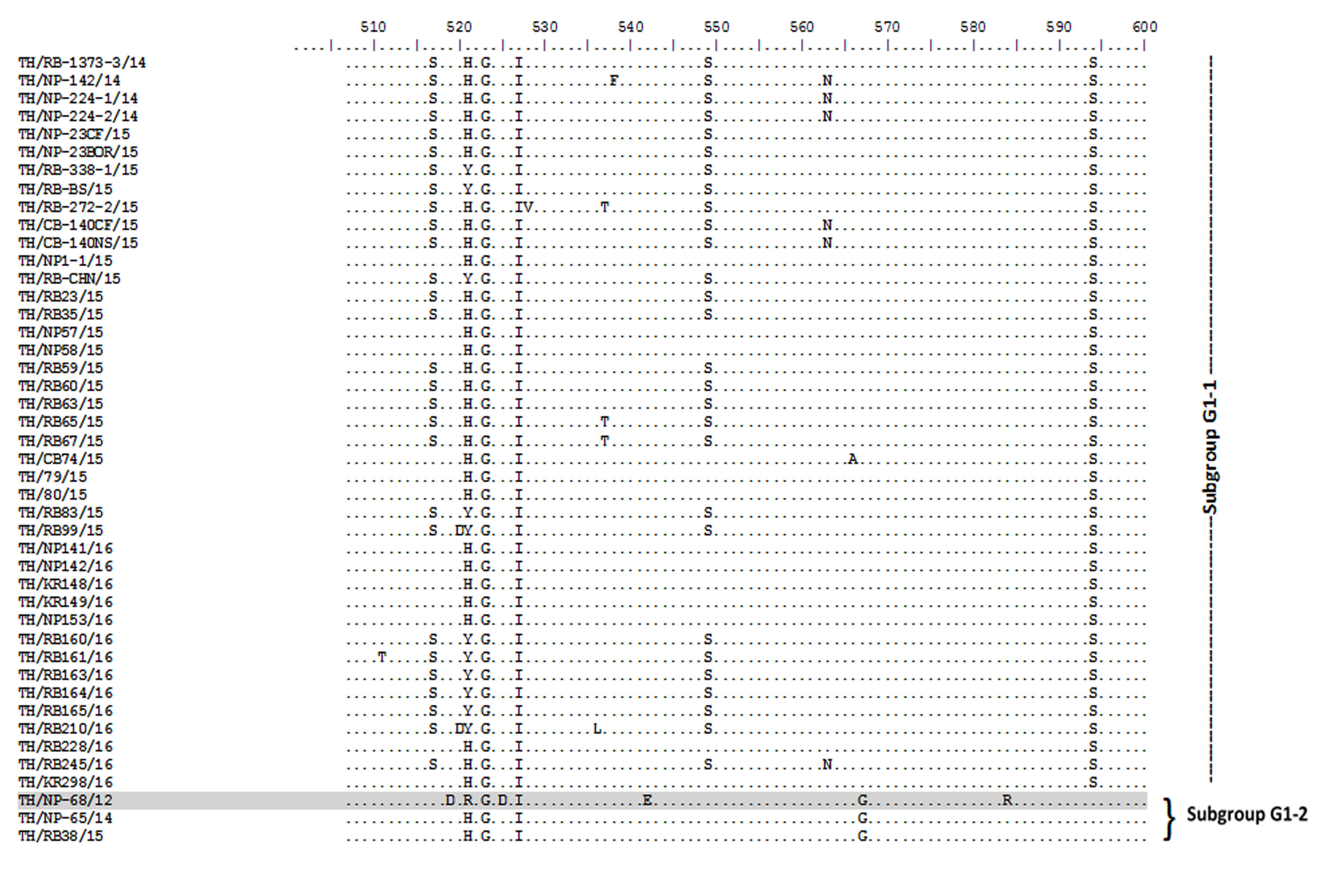

Supplement: Supplemental Information 4 — Numbers indicate residue position. Identical residues are dotted. Strain NP-68/12 differed most from CV777 which showed in gray highlight. [file peerj-07-6843-s004.png]

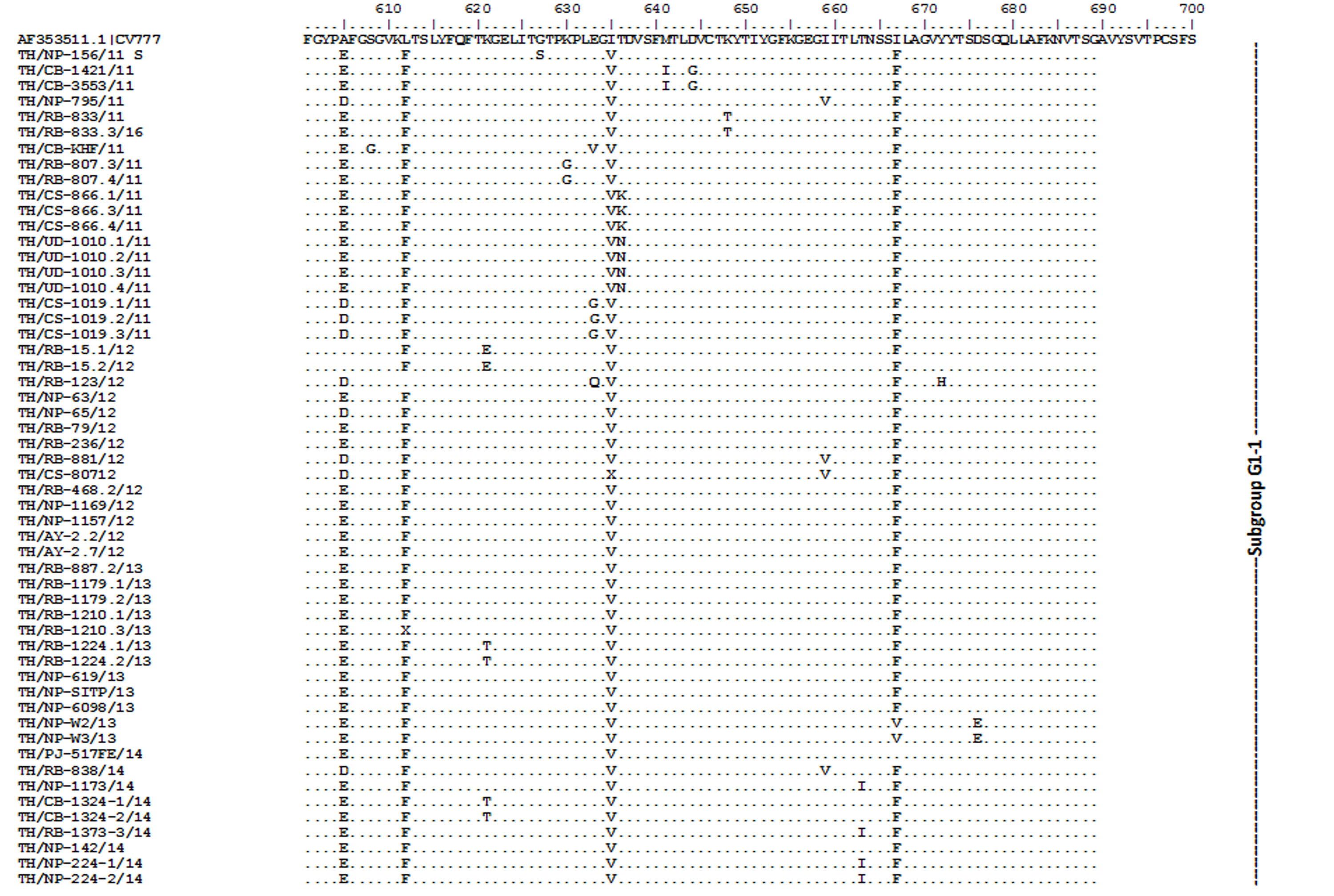

Supplement: Supplemental Information 5 — Numbers indicate residue position. Identical residues are dotted. Strain NP-68/12 differed most from CV777 which showed in gray highlight. [file peerj-07-6843-s005.png]

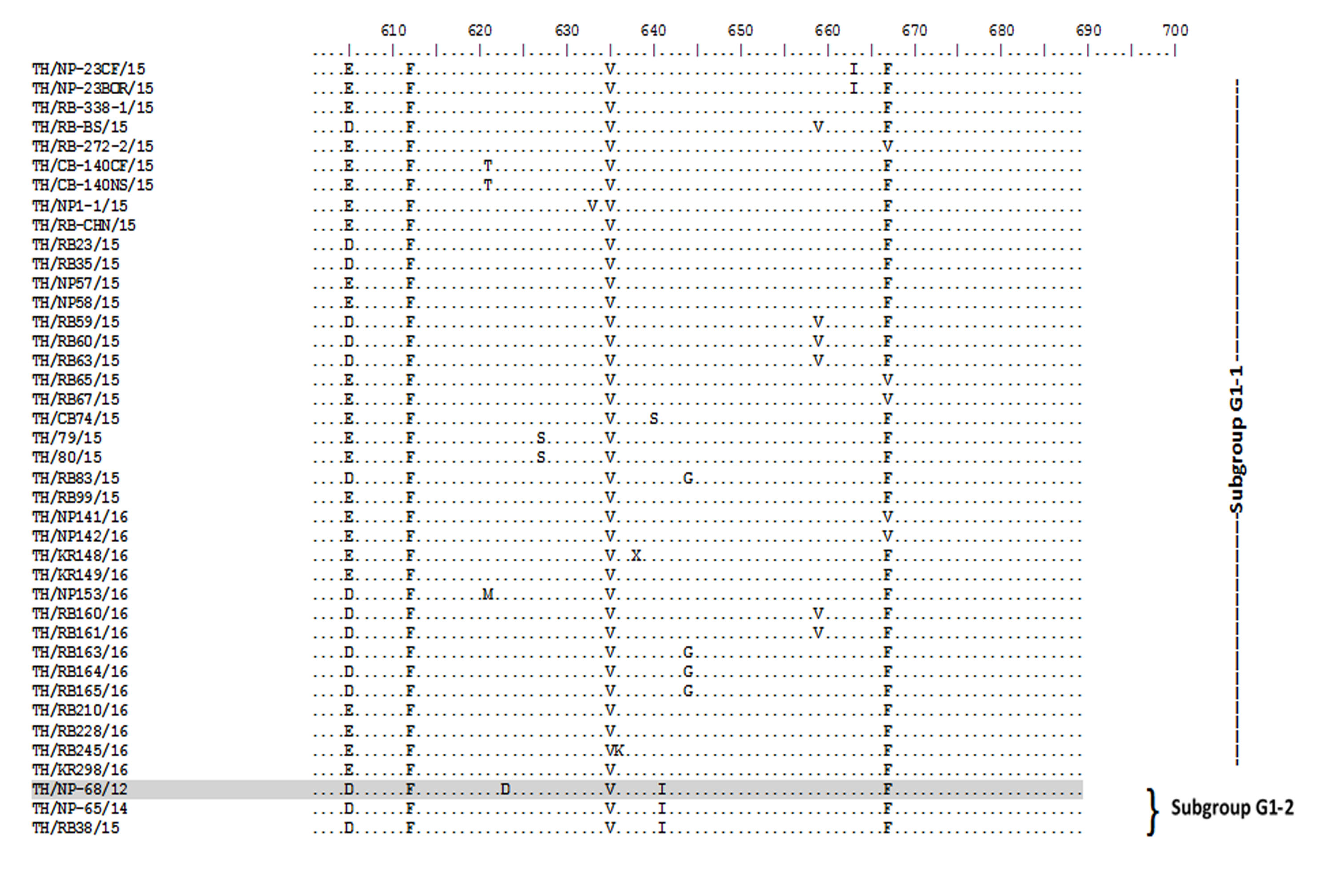

Supplement: Supplemental Information 6 — Numbers indicate residue position. Identical residues are dotted. Strain NP-68/12 differed most from CV777 which showed in gray highlight. [file peerj-07-6843-s006.png]

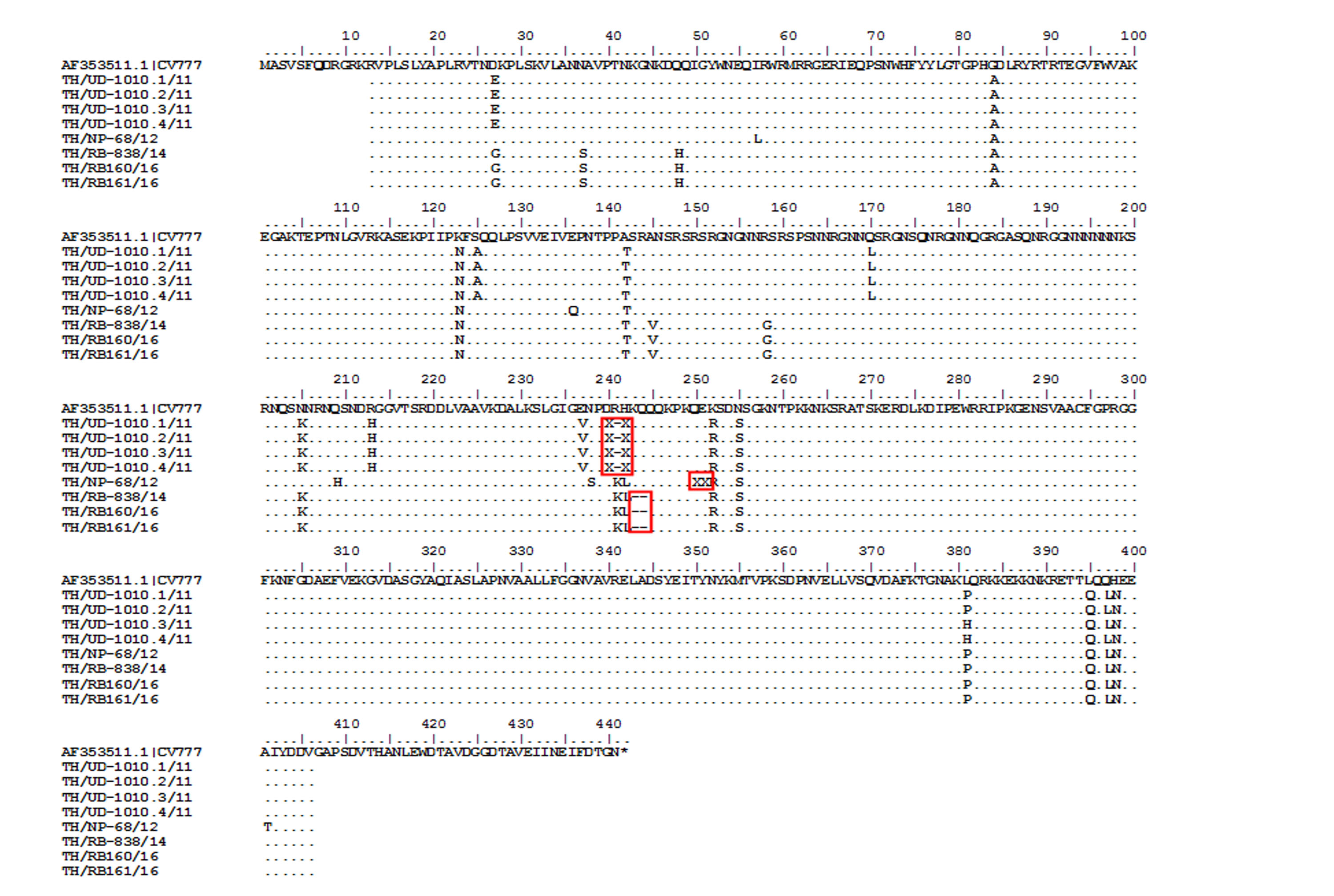

Supplement: Supplemental Information 7 — Numbers indicate residue position. Identical residues are dotted. Deletions are noted with dashed lines covered by the red boxes. [file peerj-07-6843-s007.png]
