# Supplementary material for: Analysis of the spike, ORF3, and nucleocapsid genes of porcine epidemic diarrhea virus circulating on Thai swine farms, 2011–2016
Source: PeerJ. 2019 Apr 30;7:e6843. doi: 10.7717/peerj.6843 (PMC6499054; doi:10.7717/peerj.6843)
Supplement: Supplemental Information 8 — Nucleotide primers used to amplify the PEDV genes. [file peerj-07-6843-s008.docx]

|  | **Sequences** | **Annealing temperature** | **Location** | **PCR product size** |
| --- | --- | --- | --- | --- |
| **S gene** | 5’-TTCTGAGTCACGAACAGCCA-3’ | 55°C | 1466-1485 | 651 bp |
| (Kim et al. 2001) | 5’-CATATGCAGCCTGCTCTGAA-3’ |  | 2097-2116 |  |
| **N gene** | 5’-CTAAACAGAAACTTTATGGCTT-3’ | 55°C | 79-100 | 760 bp |
| (DQ355223.1) | 5’-ATGTCTTTGAGGTCACGTTC-3’ |  | 907-926 |  |
|  | 5’-CTTCTCAGAACAGAGGAGG-3’ | 55°C | 650-668 | 848 bp |
|  | 5’-GTGTCACCACCATCAACAG-3’ |  | 1358-1376 |  |
| **ORF3 gene** | 5'-CGAAGCTTTTGAAAAGGTCC-3' | 51°C | 18-37 | 755 bp |
| (GU372734.1) | 5'-GGAAAAAGAGTACGAAAAGCC-3' |  | 752-772 |  |
