# Supplementary material for: Analysis of the spike, ORF3, and nucleocapsid genes of porcine epidemic diarrhea virus circulating on Thai swine farms, 2011–2016
Source: PeerJ. 2019 Apr 30;7:e6843. doi: 10.7717/peerj.6843 (PMC6499054; doi:10.7717/peerj.6843)
Supplement: Supplemental Information 9 [file peerj-07-6843-s009.docx]

|  |  | **Accession number** |  |
| --- | --- | --- | --- |
| **Strain name** | **S** | **ORF3** | **N** |
| **TH/NP-156/11** | KX911490 | MH744158 | MH744261 |
| **TH/CB-1421/11** | KX911491 | MH744159 | MH744262 |
| **TH/CB-3553/11** | KX911492 | MH744160 | MH744263 |
| **TH/NP-795/11** | KX911493 | MH744161 | MH744264 |
| **TH/RB-833/11** | KX911494 | MH744162 | MH744265 |
| **TH/RB-833.3/11** | MF139512 | MH744163 | MH744266 |
| **TH/CB-KHF/11** | KX911495 | MH744164 | MH744267 |
| **TH/RB-807.3/11** | KX911496 | MH744165 | MH744268 |
| **TH/RB-807.4/11** | KX911497 | MH744166 | MH744269 |
| **TH/CS-866.1/11** | KX911498 | MH744167 | MH744270 |
| **TH/CS-866.3/11** | KX911499 | MH744168 | MH744271 |
| **TH/CS-866.4/11** | KX911500 | MH744169 | MH744272 |
| **TH/UD-1010.1/11** | KX911501 | MH744170 | MH744253 |
| **TH/UD-1010.2/11** | KX911502 | MH744171 | MH744254 |
| **TH/UD-1010.3/11** | KX911503 | MH744172 | MH744255 |
| **TH/UD-1010.4/11** | KX911504 | MH744173 | MH744256 |
| **TH/CS-1019.1/11** | KX911505 | MH744174 | MH744273 |
| **TH/CS-1019.2/11** | KX911506 | MH744175 | MH744274 |
| **TH/CS-1019.3/11** | KX911507 | MH744176 | MH744275 |
| **TH/RB-15.1/12** | KX911508 | MH744177 | MH744276 |
| **TH/RB-15.2/12** | KX911509 | MH744178 | MH744277 |
| **TH/NP-68/12** | KX911510 | MH744179 | MH744260 |
| **TH/RB-123/12** | MF139511 | MH744180 | MH744278 |
| **TH/NP-63/12** | KX911511 | MH744181 | MH744279 |
| **TH/NP-65/12** | KC858259 | MH744182 | MH744280 |
| **TH/RB-79/12** | KC858260 | MH744183 | MH744281 |
| **TH/RB-236/12** | KX911512 | MH744184 | MH744282 |
| **TH/RB-881/12** | KX911513 | MH744185 | MH744283 |
| **TH/CS-80712** | KX911514 | MH744186 | MH744284 |
| **TH/RB-468.2/12** | KX911515 | MH744187 | MH744285 |
| **TH/NP-1169/12** | KX911517 | MH744188 | MH744286 |
| **TH/NP-1157/12** | KX911516 | MH744189 | MH744287 |
| **TH/AY-2.2/12** | KC858261 | MH744190 | MH744288 |
| **TH/AY-2.7/12** | KC858262 | MH744191 | MH744289 |
| **TH/RB-887.2/13** | KX911518 | MH744192 | MH744290 |
| **TH/RB-1179.1/13** | KX911519 | MH744193 | MH744291 |
| **TH/RB-1179.2/13** | KX911520 | MH744194 | MH744292 |
| **TH/RB-1210.1/13** | KX911521 | MH744195 | MH744293 |
| **TH/RB-1210.3/13** | KX911522 | MH744196 | MH744294 |
| **TH/RB-1224.1/13** | KX911523 | MH744197 | MH744295 |
| **TH/RB-1224.2/13** | KX911524 | MH744198 | MH744296 |
| **TH/NP-619/13** | KX911526 | MH744199 | MH744297 |
| **TH/NP-SITP/13** | KX911527 | MH744200 | MH744298 |
| **TH/NP-6098/13** | KX911528 | MH744201 | MH744299 |
| **TH/NP-W2/13** | KX911529 | MH744202 | MH744300 |
| **TH/NP-W3/13** | KX911530 | MH744203 | MH744301 |
| **TH/PJ-517FE/14** | KX911532 | MH744204 | MH744302 |
| **TH/RB-838/14** | KX911533 | MH744205 | MH744257 |
| **TH/NP-1173/14** | KX911543 | MH744206 | MH744303 |
| **TH/CB-1324-1/14** | KX911544 | MH744207 | MH744304 |
| **TH/CB-1324-2/14** | KX911545 | MH744208 | MH744305 |
| **TH/RB-1373-3/14** | KX911546 | MH744209 | MH744306 |
| **TH/NP-65/14** | KX911548 | MH744210 | MH744307 |
| **TH/NP-142/14** | KX911550 | MH744211 | MH744308 |
| **TH/NP-224-1/14** | KX911551 | MH744212 | MH744309 |
| **TH/NP-224-2/14** | KX911552 | MH744213 | MH744310 |
| **TH/NP-23CF/15** | KX911554 | MH744214 | MH744311 |
| **TH/NP-23BOR/15** | KX911555 | MH744215 | MH744312 |
| **TH/RB-338-1/15** | KX911556 | MH744216 | MH744313 |
| **TH/RB-BS/15** | KX911559 | MH744217 | MH744314 |
| **TH/RB-272-2/15** | KX911561 | MH744218 | MH744315 |
| **TH/CB-140CF/15** | KX911562 | MH744219 | MH744316 |
| **TH/CB-140NS/15** | KX911563 | MH744220 | MH744317 |
| **TH/NP1-1/15** | KX911564 | MH744221 | MH744318 |
| **TH/RB-CHN/15** | KX911567 | MH744222 | MH744319 |
| **TH/RB23/15** | KX911568 | MH744223 | MH744320 |
| **TH/RB35/15** | KX911570 | MH744224 | MH744321 |
| **TH/RB38/15** | KX911571 | MH744225 | MH744322 |
| **TH/NP57/15** | KX911573 | MH744226 | MH744323 |
| **TH/NP58/15** | KX911572 | MH744227 | MH744324 |
| **TH/RB59/15** | KX911574 | MH744228 | MH744325 |
| **TH/RB60/15** | KX911575 | MH744229 | MH744326 |
| **TH/RB63/15** | KX911577 | MH744230 | MH744327 |
| **TH/RB65/15** | KX911579 | MH744231 | MH744328 |
| **TH/RB67/15** | KX911581 | MH744232 | MH744329 |
| **TH/CB74/15** | KX911584 | MH744233 | MH744330 |
| **TH/79/15** | KX911585 | MH744234 | MH744331 |
| **TH/80/15** | KX911586 | MH744235 | MH744332 |
| **TH/RB83/15** | KX911587 | MH744236 | MH744333 |
| **TH/RB84/15** | KX911588 | MH744237 | MH744334 |
| **TH/RB99/16** | KX911591 | MH744238 | MH744335 |
| **TH/NP141/16** | KX911594 | MH744239 | MH744336 |
| **TH/NP142/16** | KX911595 | MH744240 | MH744337 |
| **TH/KR148/16** | KX911596 | MH744241 | MH744338 |
| **TH/KR149/16** | KX911597 | MH744242 | MH744339 |
| **TH/NP153/16** | KX911598 | MH744243 | MH744340 |
| **TH/RB160/16** | KX911599 | MH744244 | MH744258 |
| **TH/RB161/16** | KX911600 | MH744245 | MH744259 |
| **TH/RB163/16** | KX911601 | MH744246 | MH744341 |
| **TH/RB164/16** | KX911602 | MH744247 | MH744342 |
| **TH/RB165/16** | KX911603 | MH744248 | MH744343 |
| **TH/RB210/16** | MF139376 | MH744249 | MH744344 |
| **TH/RB228/16** | MF139377 | MH744250 | MH744345 |
| **TH/RB245/16** | MF139383 | MH744251 | MH744346 |
