# Supplementary material for: Analysis of the spike, ORF3, and nucleocapsid genes of porcine epidemic diarrhea virus circulating on Thai swine farms, 2011–2016
Source: PeerJ. 2019 Apr 30;7:e6843. doi: 10.7717/peerj.6843 (PMC6499054; doi:10.7717/peerj.6843)
Supplement: Supplemental Information 10 — NP, Nakorn Pathom; RB, Ratchaburi; CS, Chachoengsao; CB, Chonburi; UD, Udon Thani; AY, Phra Nakhon Si Ayutthaya; PJ, Prachuap Khiri Khan; NR, Nakhon Ratchasima; and n/a, unknown farm location. [file peerj-07-6843-s010.docx]

|  | **2011 (n=19)** | **2012 (n=15)** | **2013 (n=12)** | **2014 (n=12)** | **2015 (n=22)** | **2016 (n=15)** |
| --- | --- | --- | --- | --- | --- | --- |
| **NP** | TH/NP-156/11 | TH/NP-68/12 | TH/NP-SITP/13 | TH/NP-65/14 | TH/NP-23CF/15 | TH/NP141/16 |
| **(n=25)** | TH/NP-795/11 | TH/NP-63/12 | TH/NP-6098/13 | TH/NP-142/14 | TH/NP-23BOR/15 | TH/NP142/16 |
|  |  | TH/NP-1169/12 | TH/NP-W2/13 | TH/NP-224-1/14 | TH/NP1-1/15 | TH/NP153/16 |
|  |  | TH/NP-1157/12 | TH/NP-W3/13 | TH/NP-224-2/14 | TH/NP57/15 |  |
|  |  | TH/NP-65/12 | TH/NP-619/13 | TH/NP-1173/14 | TH/NP58/15 |  |
| **RB** | TH/RB-833/11 | TH/RB-15.1/12 | TH/RB-887.2/13 | TH/RB-838/14 | TH/RB-BS/15 | TH/RB99/16 |
| **(n=43)** | TH/RB-833.3/11 | TH/RB-15.2/12 | TH/RB-1179.1/13 | TH/RB-1373-3/14 | TH/RB-CHN/15 | TH/RB160/16 |
|  | TH/RB-807.3/11 | TH/RB-123/12 | TH/RB-1179.2/13 | TH/RB-338-1/15 | TH/RB23/15 | TH/RB161/16 |
|  | TH/RB-807.4/11 | TH/RB-79/12 | TH/RB-1210.1/13 | TH/RB-272-2/15 | TH/RB35/15 | TH/RB163/16 |
|  |  | TH/RB-236/12 | TH/RB-1210.3/13 |  | TH/RB38/15 | TH/RB164/16 |
|  |  | TH/RB-468.2/12 | TH/RB-1224.1/13 |  | TH/RB59/15 | TH/RB165/16 |
|  |  | TH/RB-881/12 | TH/RB-1224.2/13 |  | TH/RB60/15 | TH/RB210/16 |
|  |  |  |  |  |  | TH/RB228/16 |
|  |  |  |  |  |  | TH/RB245/16 |
| **CS** | TH/CS-1019.1/11 | TH/CS-80712/12 |  |  |  |  |
| **(n=7)** | TH/CS-1019.2/11 |  |  |  |  |  |
|  | TH/CS-1019.3/11 |  |  |  |  |  |
|  | TH/CS-866.1/11 |  |  |  |  |  |
|  | TH/CS-866.3/11 |  |  |  |  |  |
|  | TH/CS-866.4/11 |  |  |  |  |  |
| **CB** | TH/CB-1421/11 |  |  | TH/CB-1324-1/14 | TH/CB-140CF/15 |  |
| **(n=8)** | TH/CB-3553/11 |  |  | TH/CB-1324-2/14 | TH/CB-140NS/15 |  |
|  | TH/CB-KHF/11 |  |  |  | TH/CB74/15 |  |

|  | **2011 (n=19)** | **2012 (n=15)** | **2013 (n=12)** | **2014 (n=12)** | **2015 (n=22)** | **2016 (n=15)** |
| --- | --- | --- | --- | --- | --- | --- |
| **UD** | TH/UD-1010.1/11 |  |  |  |  |  |
| **(n=4)** | TH/UD-1010.2/11 |  |  |  |  |  |
|  | TH/UD-1010.3/11 |  |  |  |  |  |
|  | TH/UD-1010.4/11 |  |  |  |  |  |
| **AY** |  | TH/AY-2.2/12 |  |  |  |  |
| **(n=2)** |  | TH/AY-2.7/12 |  |  |  |  |
| **PJ** |  |  |  | TH/PJ-517FE/14 |  |  |
| (n=1) |  |  |  |  |  |  |
| **NR** |  |  |  |  |  | TH/KR148/16 |
| **(n=3)** |  |  |  |  |  | TH/KR149/16 |
|  |  |  |  |  |  | TH/KR298/16 |
| **n/a** |  |  |  |  | TH/79/15 |  |
| (n=2) |  |  |  |  | TH/80/15 |  |

**continued*
