# Supplementary material for: Analysis of the spike, ORF3, and nucleocapsid genes of porcine epidemic diarrhea virus circulating on Thai swine farms, 2011–2016
Source: PeerJ. 2019 Apr 30;7:e6843. doi: 10.7717/peerj.6843 (PMC6499054; doi:10.7717/peerj.6843)
Supplement: Supplemental Information 11 — The COE domain of Thai PEDVs had the most sequence variation compared to vaccine strains, while ORF3 and N genes are more conserved. [file peerj-07-6843-s011.docx]

|  | **Amino acid identity of Thai PEDV strains** | | |
| --- | --- | --- | --- |
| **Vaccine strains** | **S gene (COE domain)** | **ORF3 gene** | **N gene** |
| **Attenuated DR13** | 94.8-98.5% | 96.7-98.1% | 95.1-97% |
| **83P-5** | 94.8-98.5% | n/a | 95.2-97% |
| **P-5V** | 94.8-98.5% | 96.7-98.1% | n/a |
| **KPEDV-9** | 93.7-97.2% | n/a | n/a |
| **94P4C6** | 95.8-97% | 95-96.5% | 94.5-96.3% |
| **CV777** | 92.8-96.6% | 95.8-97% | 94.3-95.7% |

n/a = sequence not available
