# Supplementary material for: Analysis of the spike, ORF3, and nucleocapsid genes of porcine epidemic diarrhea virus circulating on Thai swine farms, 2011–2016
Source: PeerJ. 2019 Apr 30;7:e6843. doi: 10.7717/peerj.6843 (PMC6499054; doi:10.7717/peerj.6843)
Supplement: Supplemental Information 12 — Vaccine strains include Attenuated DR13, 94P4-C6, and P-5V (strain KPEDV-9, 83P-5 were excluded because their sequence not available). [file peerj-07-6843-s012.docx]

|  |  |  | **Position of amino acid point mutations in each gene** | | | | | |  |  |  |  |  |  |  |
| --- | --- | --- | --- | --- | --- | --- | --- | --- | --- | --- | --- | --- | --- | --- | --- |
|  | **S (COE domain)** |  |  |  |  | **ORF3** |  |  |  |  |  | **N gene** |  |  |  |
| **aa position** | **517** | **549** | **594** |  | **82-98** | **138-139** | **165** | **182** |  | **123** | **142** | **145** | **242** | **380** | **397** |
| **Thai PEDV strains** | S | S | S |  | YCPLLYYCGAFLDATIIC | YY | V | Q/H |  | N | T | A/V | L | K | L |
| **CV777** | A | T | G |  | YCPLLYYCGALLDATIIC | YY | S | H |  | K | A | A | H | K | L |
| **Other vaccine strains*** | A | T | G |  | deletion | deletion | F | H |  | K | A | T | H | I | Q |

aa= amino acid
