# Supplementary material for: Analysis of the spike, ORF3, and nucleocapsid genes of porcine epidemic diarrhea virus circulating on Thai swine farms, 2011–2016
Source: PeerJ. 2019 Apr 30;7:e6843. doi: 10.7717/peerj.6843 (PMC6499054; doi:10.7717/peerj.6843)
Supplement: Supplemental Information 13 — S gene is sub-divided into subgroup 1 and 3, subgroup 1 and 2 for N gene, whereas major groups of ORF3 gene are not sub-divided into subgroup. [file peerj-07-6843-s013.docx]

| **S** | | **ORF3** | | **N** | |
| --- | --- | --- | --- | --- | --- |
| **G1** | |  |  | **G3** |  |
| **G1-2** | **G1-3** | **G1** | **G2** | **G3-1** | **G3-2** |
| **(n=3)** | **(n=92)** | **(n=12)** | **(n=83)** | **(n=12)** | **(n=83)** |
| TH/NP-68/12 | TH/NP-156/11 | TH/NP1-1/15 | TH/NP-156/11 | TH/NP-156/11 | TH/NP1-1/15 |
| TH/NP-65/14 | TH/CB-1421/11 | TH/NP57/15 | TH/CB-1421/11 | TH/CB-1421/11 | TH/NP57/15 |
| TH/RB38/15 | TH/CB-3553/11 | TH/NP58/15 | TH/CB-3553/11 | TH/CB-3553/11 | TH/NP58/15 |
|  | TH/NP-795/11 | TH/CB74/15 | TH/NP-795/11 | TH/NP-795/11 | TH/CB74/15 |
|  | TH/RB-833/11 | TH/79/15 | TH/RB-833/11 | TH/RB-833/11 | TH/79/15 |
|  | TH/RB-833.3/11 | TH/80/15 | TH/RB-833.3/11 | TH/RB-833.3/11 | TH/80/15 |
|  | TH/CB-KHF/11 | TH/NP141/16 | TH/CB-KHF/11 | TH/CB-KHF/11 | TH/NP141/16 |
|  | TH/RB-807.3/11 | TH/NP142/16 | TH/RB-807.3/11 | TH/RB-807.3/11 | TH/NP142/16 |
|  | TH/RB-807.4/11 | TH/KR148/16 | TH/RB-807.4/11 | TH/RB-807.4/11 | TH/KR148/16 |
|  | TH/CS-866.1/11 | TH/KR149/16 | TH/CS-866.1/11 | TH/CS-866.1/11 | TH/KR149/16 |
|  | TH/CS-866.3/11 | TH/NP153/16 | TH/CS-866.3/11 | TH/CS-866.3/11 | TH/NP153/16 |
|  | TH/CS-866.4/11 | TH/RB228/16 | TH/CS-866.4/11 | TH/CS-866.4/11 | TH/RB228/16 |
|  | TH/UD-1010.1/11 |  | TH/UD-1010.1/11 | TH/UD-1010.1/11 |  |
|  | TH/UD-1010.2/11 |  | TH/UD-1010.2/11 | TH/UD-1010.2/11 |  |
|  | TH/UD-1010.3/11 |  | TH/UD-1010.3/11 | TH/UD-1010.3/11 |  |
|  | TH/UD-1010.4/11 |  | TH/UD-1010.4/11 | TH/UD-1010.4/11 |  |
|  | TH/CS-1019.1/11 |  | TH/CS-1019.1/11 | TH/CS-1019.1/11 |  |
|  | TH/CS-1019.2/11 |  | TH/CS-1019.2/11 | TH/CS-1019.2/11 |  |
|  | TH/CS-1019.3/11 |  | TH/CS-1019.3/11 | TH/CS-1019.3/11 |  |
|  | TH/RB-15.1/12 |  | TH/RB-15.1/12 | TH/RB-15.1/12 |  |
|  | TH/RB-15.2/12 |  | TH/RB-15.2/12 | TH/RB-15.2/12 |  |
|  | TH/RB-123/12 |  | TH/NP-68/12 | TH/NP-68/12 |  |
|  | TH/NP-63/12 |  | TH/RB-123/12 | TH/RB-123/12 |  |
|  | TH/NP-65/12 |  | TH/NP-63/12 | TH/NP-63/12 |  |
|  | TH/RB-79/12 |  | TH/NP-65/12 | TH/NP-65/12 |  |
|  | TH/RB-236/12 |  | TH/RB-79/12 | TH/RB-79/12 |  |
|  | TH/RB-881/12 |  | TH/RB-236/12 | TH/RB-236/12 |  |
|  | TH/CS-80712 |  | TH/RB-881/12 | TH/RB-881/12 |  |
|  | TH/RB-468.2/12 |  | TH/CS-80712 | TH/CS-80712 |  |
|  | TH/NP-1169/12 |  | TH/RB-468.2/12 | TH/RB-468.2/12 |  |
|  | TH/NP-1157/12 |  | TH/NP-1169/12 | TH/NP-1169/12 |  |
|  | TH/AY-2.2/12 |  | TH/NP-1157/12 | TH/NP-1157/12 |  |
|  | TH/AY-2.7/12 |  | TH/AY-2.2/12 | TH/AY-2.2/12 |  |
|  | TH/RB-887.2/13 |  | TH/AY-2.7/12 | TH/AY-2.7/12 |  |
|  | TH/RB-1179.1/13 |  | TH/RB-887.2/13 | TH/RB-887.2/13 |  |

**continued*

| **S** | | **ORF3** | | **N** | |
| --- | --- | --- | --- | --- | --- |
| **G1** | |  |  | **G3** |  |
| **G1-2** | **G1-3** | **G1** | **G2** | **G3-1** | **G3-2** |
| **(n=3)** | **(n=92)** | **(n=12)** | **(n=83)** | **(n=12)** | **(n=83)** |
|  | TH/RB-1179.1/13 |  | TH/RB-887.2/13 | TH/RB-887.2/13 |  |
|  | TH/RB-1179.2/13 |  | TH/RB-1179.1/13 | TH/RB-1179.1/13 |  |
|  | TH/RB-1210.1/13 |  | TH/RB-1179.2/13 | TH/RB-1179.2/13 |  |
|  | TH/RB-1210.3/13 |  | TH/RB-1210.1/13 | TH/RB-1210.1/13 |  |
|  | TH/RB-1224.1/13 |  | TH/RB-1210.3/13 | TH/RB-1210.3/13 |  |
|  | TH/RB-1224.2/13 |  | TH/RB-1224.1/13 | TH/RB-1224.1/13 |  |
|  | TH/NP-619/13 |  | TH/RB-1224.2/13 | TH/RB-1224.2/13 |  |
|  | TH/NP-SITP/13 |  | TH/NP-619/13 | TH/NP-619/13 |  |
|  | TH/NP-6098/13 |  | TH/NP-SITP/13 | TH/NP-SITP/13 |  |
|  | TH/NP-W2/13 |  | TH/NP-6098/13 | TH/NP-6098/13 |  |
|  | TH/NP-W3/13 |  | TH/NP-W2/13 | TH/NP-W2/13 |  |
|  | TH/PJ-517FE/14 |  | TH/NP-W3/13 | TH/NP-W3/13 |  |
|  | TH/RB-838/14 |  | TH/PJ-517FE/14 | TH/PJ-517FE/14 |  |
|  | TH/NP-1173/14 |  | TH/RB-838/14 | TH/RB-838/14 |  |
|  | TH/CB-1324-1/14 |  | TH/NP-1173/14 | TH/NP-1173/14 |  |
|  | TH/CB-1324-2/14 |  | TH/CB-1324-1/14 | TH/CB-1324-1/14 |  |
|  | TH/RB-1373-3/14 |  | TH/CB-1324-2/14 | TH/CB-1324-2/14 |  |
|  | TH/NP-142/14 |  | TH/RB-1373-3/14 | TH/RB-1373-3/14 |  |
|  | TH/NP-224-1/14 |  | TH/NP-65/14 | TH/NP-65/14 |  |
|  | TH/NP-224-2/14 |  | TH/NP-142/14 | TH/NP-142/14 |  |
|  | TH/NP-23CF/15 |  | TH/NP-224-1/14 | TH/NP-224-1/14 |  |
|  | TH/NP-23BOR/15 |  | TH/NP-224-2/14 | TH/NP-224-2/14 |  |
|  | TH/RB-338-1/15 |  | TH/NP-23CF/15 | TH/NP-23CF/15 |  |
|  | TH/RB-BS/15 |  | TH/NP-23BOR/15 | TH/NP-23BOR/15 |  |
|  | TH/RB-272-2/15 |  | TH/RB-338-1/15 | TH/RB-338-1/15 |  |
|  | TH/CB-140CF/15 |  | TH/RB-BS/15 | TH/RB-BS/15 |  |
|  | TH/CB-140NS/15 |  | TH/RB-272-2/15 | TH/RB-272-2/15 |  |
|  | TH/NP1-1/15 |  | TH/CB-140CF/15 | TH/CB-140CF/15 |  |
|  | TH/RB-CHN/15 |  | TH/CB-140NS/15 | TH/CB-140NS/15 |  |
|  | TH/RB23/15 |  | TH/RB-CHN/15 | TH/RB-CHN/15 |  |
|  | TH/RB35/15 |  | TH/RB23/15 | TH/RB23/15 |  |
|  | TH/NP57/15 |  | TH/RB35/15 | TH/RB35/15 |  |
|  | TH/NP58/15 |  | TH/RB38/15 | TH/RB38/15 |  |
|  | TH/RB59/15 |  | TH/RB59/15 | TH/RB59/15 |  |
|  | TH/RB60/15 |  | TH/RB60/15 | TH/RB60/15 |  |

**continued*

| **S** | | **ORF3** | | **N** | |
| --- | --- | --- | --- | --- | --- |
| **G1** | |  |  | **G3** |  |
| **G1-2** | **G1-3** | **G1** | **G2** | **G3-1** | **G3-2** |
| **(n=3)** | **(n=92)** | **(n=12)** | **(n=83)** | **(n=12)** | **(n=83)** |
|  | TH/RB63/15 |  | TH/RB63/15 | TH/RB63/15 |  |
|  | TH/RB65/15 |  | TH/RB65/15 | TH/RB65/15 |  |
|  | TH/RB67/15 |  | TH/RB67/15 | TH/RB67/15 |  |
|  | TH/CB74/15 |  | TH/RB83/15 | TH/RB83/15 |  |
|  | TH/79/15 |  | TH/RB84/15 | TH/RB84/15 |  |
|  | TH/80/15 |  | TH/RB99/16 | TH/RB99/16 |  |
|  | TH/RB83/15 |  | TH/RB160/16 | TH/RB160/16 |  |
|  | TH/RB84/15 |  | TH/RB161/16 | TH/RB161/16 |  |
|  | TH/RB99/16 |  | TH/RB163/16 | TH/RB163/16 |  |
|  | TH/NP141/16 |  | TH/RB164/16 | TH/RB164/16 |  |
|  | TH/NP142/16 |  | TH/RB165/16 | TH/RB165/16 |  |
|  | TH/KR148/16 |  | TH/RB210/16 | TH/RB210/16 |  |
|  | TH/KR149/16 |  | TH/RB245/16 | TH/RB245/16 |  |
|  | TH/NP153/16 |  | TH/KR298/16 | TH/KR298/16 |  |
|  | TH/RB160/16 |  |  |  |  |
|  | TH/RB161/16 |  |  |  |  |
|  | TH/RB163/16 |  |  |  |  |
|  | TH/RB164/16 |  |  |  |  |
|  | TH/RB165/16 |  |  |  |  |
|  | TH/RB210/16 |  |  |  |  |
|  | TH/RB228/16 |  |  |  |  |
|  | TH/RB245/16 |  |  |  |  |
